# Genetic Predisposition to Lone Atrial Fibrillation and the Causal Effect on Cardiovascular Diseases: A Mendelian Randomization Study

**DOI:** 10.3390/biomedicines14020413

**Published:** 2026-02-11

**Authors:** Seunghwan Park, Hwajung Kim, Jieun Seo, Do Young Kim, Youmi Hwang, Sung-Hwan Kim, Kichang Lee, Wonil Chung, Young Choi

**Affiliations:** 1 Department of Statistics and Actuarial Science, College of Natural Sciences, Soongsil University, Seoul 06978, Republic of Korea; shp422@soongsil.ac.kr (S.P.); jieun951228@gmail.com (J.S.); 2 Division of Cardiology, Department of Internal Medicine, Yeouido St. Mary’s Hospital, College of Medicine, The Catholic University of Korea, Seoul 06591, Republic of Korea; hjkimmmmm@gmail.com; 3 Cardiovascular Research Institute for Intractable Disease, College of Medicine, The Catholic University of Korea, Seoul 06591, Republic of Korea; ehdud0126@gmail.com (D.Y.K.); youmi0607@naver.com (Y.H.); sunghwan@amc.seoul.kr (S.-H.K.); 4 Division of Cardiology, Department of Internal Medicine, Seoul St. Mary’s Hospital, College of Medicine, The Catholic University of Korea, Seoul 06591, Republic of Korea; 5 Division of Cardiology, Department of Internal Medicine, St. Vincent’s Hospital, College of Medicine, The Catholic University of Korea, Seoul 06591, Republic of Korea; 6 Cardiovascular Research Center, Massachusetts General Hospital, Boston, MA 02114, USA; kichang.lee@mgh.harvard.edu; 7 Harvard Medical School, Boston, MA 02115, USA; 8 Program in Genetic Epidemiology and Statistical Genetics, Harvard T.H. Chan School of Public Health, Boston, MA 02115, USA

**Keywords:** atrial fibrillation, lone atrial fibrillation, Mendelian randomization, stroke, heart failure

## Abstract

**Background:** Lone atrial fibrillation (AF) is characterized by the absence of discernible risk factors, yet its long-term prognostic implications remain unclear. We evaluated genetic predisposition to lone AF and conducted a Mendelian randomization (MR) study to assess its causal effect on cardiovascular outcomes. **Methods:** A genome-wide association study (GWAS) for lone AF, along with common AF was conducted using UK Biobank data. Lone AF was defined as AF occurring without clinical risk factors. Summary-level data for cardiovascular phenotypes were obtained from publicly available GWAS datasets and the causal effects were estimated using MR. **Results:** We identified 36 single-nucleotide polymorphisms associated with lone AF, including two novel loci. In MR analyses, lone AF was significantly associated with an increased risk of stroke (odds ratio [OR] 2.62, 95% confidence interval [CI] 2.14–3.22) and heart failure (HF) (OR 2.55, 95% CI 2.14–3.04). The associations with coronary artery disease (CAD) (OR 0.90, 95% CI 0.73–1.10) and cardiac death (OR 1.32, 95% CI 0.99–1.77) were not significant. MR analyses of common AF also demonstrated significant associations with stroke (OR 1.86, 95% CI 1.69–2.04) and HF (OR 1.71, 95% CI 1.59–1.84), though the effect sizes were smaller compared to those of lone AF. **Conclusions:** Genetic predisposition to lone AF is associated with more than a twofold increase in the risk of stroke and HF. However, no clear association was observed between lone AF and CAD or cardiac death.

## 1. Introduction

Atrial fibrillation (AF) is the most common sustained cardiac arrhythmia, with a prevalence of approximately 1% in the general population [1]. AF leads to irregular cardiac contractions and blood stasis in the atria, thereby increasing the risk of heart failure (HF) and stroke [2]. AF is more prevalent in elderly patients with multiple comorbidities, and these risk factors overlap with those for stroke and HF [2,3]. Therefore, it is often challenging to isolate the risks of adverse clinical outcomes attributed solely to AF. Mendelian randomization (MR) is a statistical method used to assess causal associations through natural randomization, using each individual’s genetic predisposition as an instrumental variable [4]. Previous MR studies have shown that AF is associated with an approximately 1.2-fold increased risk of stroke and HF, but not with coronary heart disease [5,6]. However, considering that clinical studies have shown that AF increases the risk of stroke or HF by 2 to 5-fold, the effect observed in MR studies appears relatively small [7,8]. If the genetic variants used in MR studies are also associated with other cardiovascular phenotypes beyond the impact of AF, it may compromise the accuracy of the causal inference [9].

Lone AF is a subtype of AF that occurs in patients without identifiable clinical risk factors. It typically refers to early-onset isolated AF and accounts for approximately 3% to 15% of all AF cases [10]. Despite its relatively low prevalence, lone AF represents a clinically important subgroup because it affects younger individuals who otherwise lack conventional cardiovascular risk factors. Genetic factors are considered to play an important role in the development of lone AF. While pathogenic rare variants in genes associated with ion channels, electrical coupling, or cardiomyopathy are thought to have an association with lone AF, the specific role of common single-nucleotide polymorphisms (SNPs) remains poorly understood [11]. The natural course of lone AF is generally considered favorable [12]. Previous reports on the long-term clinical outcomes of lone AF have suggested that the risks of death, stroke, and HF are not significantly elevated compared to the general adult population [13,14]. However, these findings are primarily based on small-scale observational studies without suitable control groups, making it challenging to accurately isolate the effects of lone AF. Moreover, clinical studies on lone AF may overestimate the prognosis because patients with genetically predisposed, early-onset AF may not be classified as having ‘isolated AF’ if they have developed common chronic conditions at the time of analysis. Also, clinical studies may face challenges in establishing control groups, which limits the ability to accurately assess the actual risk of cardiovascular events caused solely by AF. These limitations can be addressed through MR analyses, and particularly, MR studies using genetic predisposition for lone AF may more accurately isolate the cardiovascular risks associated explicitly with AF. In this study, we performed a genome-wide association study (GWAS) to identify SNPs associated with lone AF. We then conducted MR analyses to evaluate the causal effect of lone AF on various cardiovascular phenotypes and compared these results with the effect of common forms of AF to better understand the distinct contributions of lone AF to cardiovascular risk.

## 2. Method

**Figure 1:**
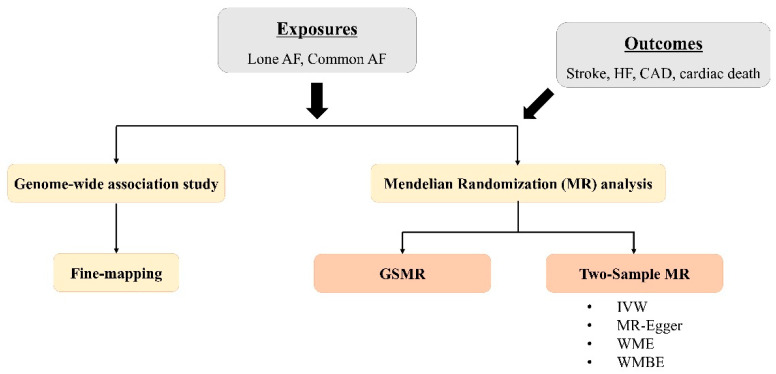
Overview of the study flow. AF = atrial fibrillation; HF = heart failure; CAD = coronary artery disease; GSMR = generalized summary-data-based MR; IVW = inverse variance weighted; WME = weighted median; WMBE = weighted mode-based estimator.

A GWAS for lone AF, along with common AF, was conducted using the UK Biobank (UKB) dataset. AF was diagnosed by the International Classification of Disease (ICD)-10 code of I48 in hospital records or 12-lead electrocardiogram (ECG) interpretation of AF. Lone AF was defined as a diagnosis of AF without coexisting conditions, including age ≥ 60 years, severe obesity, heavy alcohol consumption, hypertension, diabetes mellitus, coronary artery disease (CAD), valvular heart disease, pulmonary disease, cardiomyopathy, hyperthyroidism, or obstructive sleep apnea (Supplemental Table S1). Controls for the lone AF GWAS were defined as individuals without a diagnosis of AF, irrespective of the presence of cardiometabolic or structural risk factors. GWAS statistics were adjusted for potential confounders, including age, age squared, gender, genotype principal components (PCs), assessment array, and genotyping array using BOLT-LMM v2.3. Summary-level data for cardiovascular phenotypes were obtained from public GWAS datasets. Causal effects were estimated through MR analyses using the generalized summary-data-based MR (GSMR) method [15] as the main analysis, and two-sample MR methods were additionally performed (Figure 1). The datasets used in our analyses are summarized in Table 1. This study protocol was approved by the institutional review board of the Seoul St. Mary’s Hospital.

### 2.1. Instrumental Variables

The UKB, a large-scale prospective cohort study, recruited 503,325 participants aged 40 to 69 years from 22 UK study centers between 2006 and 2010. Our analysis specifically focused on 459,119 individuals of European ancestry with high-quality genotyping and comprehensive phenotype/covariate datasets. All information from the UKB was current up to 19 December 2022. We filtered SNPs based on minor allele frequency (MAF) > 0.01 and imputation quality > 0.8, resulting in a final set of 9,572,559 SNPs used. In our analysis, we employed the clumping method in PLINK to select SNPs linked to traits from GWAS. We performed a clumping procedure to refine the set of significant SNPs, setting a linkage disequilibrium (LD) threshold of r^2^ < 0.1 and a *p*-value threshold of <5 × 10^−8^, using the 1000 Genome Project reference panel of the European population. Within a 1000 kb window, we retained the independent SNPs with the lowest *p*-values.

### 2.2. Summary Data Sources for Outcome Analyses

For stroke data, we utilized the dataset provided by the Meta-analysis of Genome-wide Association Studies of Stroke (MEGASTROKE) consortium, which is an international collaborative project aimed at studying the genetic factors of ischemic stroke through large-scale GWAS [16]. This dataset comprises 7,977,647 SNPs based on MAF ≥ 0.01 and imputation R^2^ > 0.5 and includes 40,585 cases and 406,111 controls. We utilized two sets of summary statistics for HF. First, we used GWAS summary statistics data from 26 studies within the Heterogeneity and Remission of Metabolic Syndrome (HERMES) Consortium, which includes 47,309 HF patients and 930,014 controls of European ancestry and comprises 8,281,262 SNPs based on MAF > 0.01 and imputation R^2^ > 0.5 [17]. Second, we used multi-ancestry HF GWAS data from the Global Biobank Meta-analysis Initiative (GBMI), which includes 68,408 HF cases and 1,286,331 controls and comprises 33,813,931 SNPs based on MAF > 0.01 and imputation R^2^ > 0.3 [18]. We downloaded CAD datasets provided by the Coronary Artery Disease Genome-Wide Replication and Meta-Analysis Plus C4D (CARDIoGRAMplusC4D) consortium, which comprises a meta-analysis of 48 studies, including 60,801 cases and 123,504 controls of European, South Asian, and East Asian descent [19]. This dataset consists of 9,455,778 SNPs based on MAF > 0.005 and imputation R^2^ > 0.9. We downloaded and analyzed the cardiac death data from release 7 of the FinnGen [20]. This dataset includes 13,673 cases and 295,481 controls with 15,774,061 SNPs based on MAF > 0.0001, and imputation R^2^ > 0.6. The details of the summary datasets are presented in Table 1. For all MR analyses, we restricted outcome GWAS summary statistics to individuals of European ancestry to ensure ancestry matching with the exposure GWAS conducted in the UKB. In addition, although one outcome consortium includes partial overlap with UKB participants, this overlap is limited, and given the large sample size and strong instrument strength, any resulting bias is expected to be minimal [21].

### 2.3. MR Analysis

To investigate potential causal relationships between lone AF, common AF, and cardiovascular comorbidities, we conducted an instrumental variable analysis using MR implemented in GSMR. GSMR applies stringent criteria to select independent SNP instruments and extends conventional MR by considering the sampling variance in the genetic effects on both exposure ($b_{\mathrm{zx}}$) and outcome ($b_{\mathrm{zy}}$) when estimating the instrumental effect. Pleiotropy is a major source of bias in MR analyses, potentially leading to distorted causal estimates and inflated false-positive rates. To address this, we applied the heterogeneity in dependent instruments (HEIDI) test (*p* < 0.01) within GSMR to exclude pleiotropic SNPs from the analysis. As a sensitivity analysis, two-sample MR [22] analyses were performed using SNPs obtained from the GSMR results. Heterogeneity between causal estimates was first assessed using the MR-Pleiotropy Residual Sum and Outlier (MR-PRESSO) [23] global test and Cochran’s Q statistic. When the MR-PRESSO global test yielded a significant *p*-value (<0.05), indicating substantial heterogeneity, we then applied the MR–PRESSO outlier test to remove heterogeneous outliers and obtain pleiotropy-corrected estimates. We applied multiple two-sample MR methods, including inverse variance weighted (IVW), MR-Egger [24], weighted median (WME) [25], and weighted mode-based estimator (WMBE) [26]. Multiple testing across outcomes was controlled using the false discovery rate (FDR) method, with statistical significance defined as FDR < 0.05.

## 3. Result

### 3.1. GWAS Results

**Figure 2:**
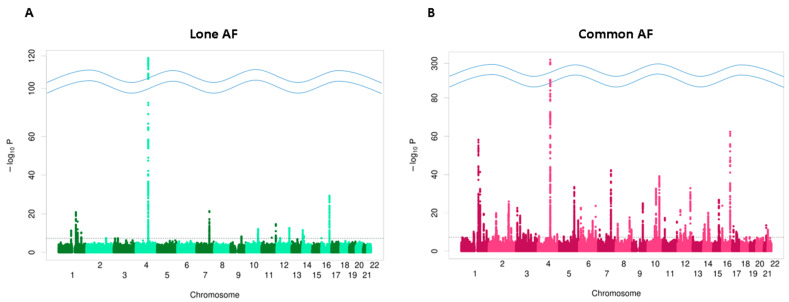
Manhattan plot of the genome-wide association study (GWAS) results in the UKB dataset. The plot shows genetic loci associated with lone atrial fibrillation (AF) (**A**) and common AF (**B**) at a significance level of *p* < 5 × 10^−8^. The horizontal dotted line indicates the genome-wide significance threshold (*p* < 5 × 10^−8^). The curved lines indicate a break in the y-axis scale, implemented to accommodate highly significant peaks with extremely small *p*-value.

Using the UKB dataset, we identified 40,203 subjects with AF and 417,589 subjects without AF. After excluding patients with clinical AF risk factors, 4767 subjects were classified as having lone AF. The results of the GWAS for lone AF and common AF are displayed in Figure 2. The GWAS identified 36 SNPs that exceeded the significant threshold for lone AF and 198 SNPs for common AF, with F-statistics of 50.2 and 43.8, respectively. Of the 36 SNPs associated with lone AF, two had not been previously reported in association with AF (Table 2). Of those, one was an intron variant of chromosome 11q13, near the gene FAT3, and the other was an intron variant of the PKP2 gene on chromosome 12q13. Detailed information for the 36 independent SNPs used as genetic instruments for lone AF, including GWAS summary statistics, SNP-level F-statistics, the proportion of variance explained (R^2^), and annotation of previously reported versus novel AF loci, is provided in Supplemental Table S2. The overall SNP-based heritability estimate was 0.131 (95% confidence interval [CI]: 0.105–0.157) for lone AF and 0.143 (95% CI: 0.126–0.160) for common AF.

### 3.2. The Causal Effect of Lone AF on Cardiovascular Outcomes

**Figure 3:**
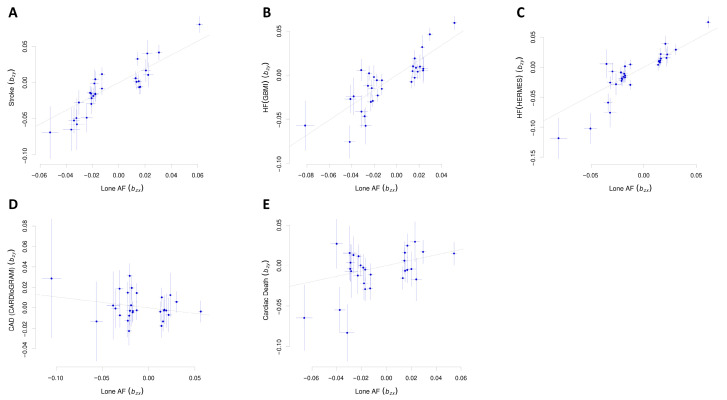
Scatter plots for generalized summary-data-based Mendelian randomization results showing the causal effect of lone atrial fibrillation (AF) on stroke (**A**), heart failure (HF) in Global Biobank Meta-Analysis Initiative (GBMI) summary data (**B**), and HF in Heterogeneity and Remission of Metabolic Syndrome (HERMES) summary data (**C**), coronary artery disease (CAD) (**D**), and cardiac death (**E**).

**Figure 4:**
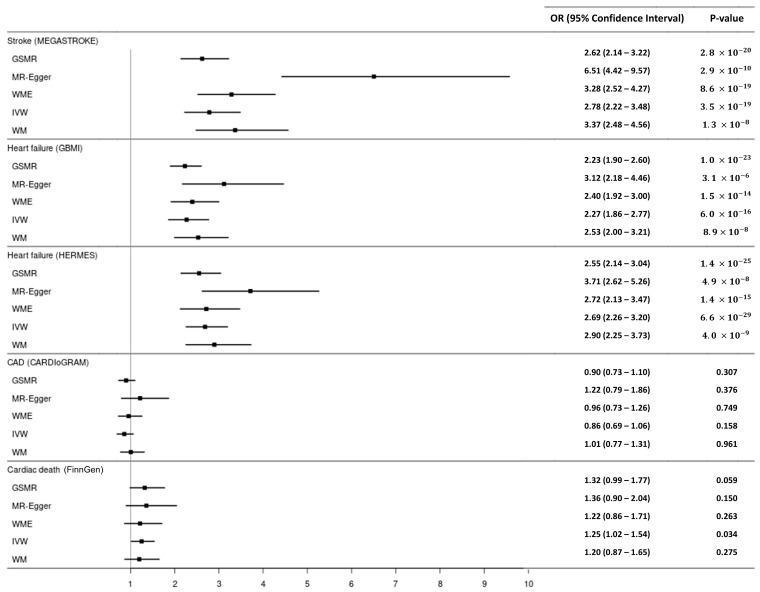
Causal effect of lone atrial fibrillation: GSMR and two-sample MR results. MR = Mendelian randomization; GSMR = generalized summary-based MR; WME = weighted median; CAD = coronary artery disease; and OR = odds ratio.

In the GSMR analysis, genetically predicted lone AF was significantly associated with an increased risk of stroke (odds ratio [OR]: 2.62, 95% CI: 2.14–3.22, *p* = 2.8 × 10^−23^) (Table 3, Figure 3), after excluding one SNP identified as a pleiotropic outlier by the HEIDI test. Lone AF was also associated with an increased risk of HF in analyses using both the GBMI (OR: 2.23, 95% CI: 1.90–2.60, *p* = 1.0 × 10^−23^), with no SNPs excluded by HEIDI-outlier filtering, and HERMES (OR: 2.55, 95% CI: 2.14–3.04, *p* = 1.4 × 10^−25^), after exclusion of one SNP identified as a pleiotropic outlier (Table 3, Figure 3). Sensitivity analyses using MR-Egger, WME, IVW, and WMBE methods confirmed the positive association between lone AF and both stroke and HF (Figure 4). These associations remained statistically significant after correction for multiple testing using the FDR.

GSMR analysis showed no significant causal effect of lone AF on CAD (OR: 0.90, 95% CI: 0.73–1.10, *p* = 0.307) (Table 3, Figure 3). Similarly, lone AF was not significantly associated with the risk of cardiac death (OR: 1.32, 95% CI: 0.99–1.77, *p* = 0.059) (Table 3, Figure 3). The sensitivity analysis results were overall consistent with the GSMR findings, except that the relationship between lone AF and cardiac death showed borderline statistical significance in the IVW method (OR: 1.25, 95% CI: 1.02–1.54, *p* = 0.034) (Figure 4, Supplemental Table S3).

### 3.3. The Causal Effect of Common AF on Cardiovascular Outcomes

In the GSMR analyses, common AF was significantly associated with elevated risks of stroke (OR: 1.86, 95% CI: 1.69–2.04, *p* = 1.5 × 10^−36^), and HF (GBMI—OR: 1.71, 95% CI: 1.59–1.84, *p* = 4.0 × 10^−48^, HERMES—OR: 1.94, 95% CI: 1.79–2.11, *p* = 3.6 × 10^−57^) (Table 3, Supplemental Figure S1). However, the estimated risk ratios for common AF with respect to stroke and HF were lower than those observed for lone AF. Sensitivity analyses using two-sample MR methods yielded consistent results (Supplemental Figure S2). In the GSMR and two-sample MR analyses, no significant association was observed between common AF and the risk of CAD (OR: 1.01, 95% CI: 0.92–1.12, *p* = 0.802) (Table 3, Supplemental Figure S2). In the GSMR analysis, common AF was significantly associated with an increased risk of cardiac death (OR: 1.28, 95% CI: 1.12–1.46, *p* = 3 × 10^−4^), unlike the results of lone AF. However, sensitivity analyses yielded inconsistent results, with a statistically significant association between common AF and cardiac death observed only in the IVW method (Supplemental Figure S2, Table S4).

## 4. Discussion

We performed a GWAS for lone AF and assessed its causal effect on cardiovascular outcomes through MR analysis. Using the UKB data, we identified 36 SNPs associated with lone AF, including two novel genetic loci not previously reported. In MR analysis, genetic predisposition to lone AF was associated with an approximately two-fold increased risk of stroke and HF, a risk level higher than that observed for common forms of AF. In contrast, lone AF did not show a clear association with CAD or cardiac death. The GSMR analysis for common AF suggested an increased risk of cardiac death; however, this finding was not consistently replicated in the sensitivity analyses.

In interpreting these MR findings, lone AF was modeled as a binary exposure under a liability-threshold framework, such that the estimated effects reflect changes in underlying susceptibility rather than a direct comparison between individuals with and without lone AF [27]. Although MR effect estimates for binary outcomes are presented on the odds ratio scale for ease of interpretation, they should be understood as scaled causal effects on an underlying continuous liability rather than causal risk ratios. While odds ratios are non-collapsible, particularly for more prevalent outcomes such as HF, this primarily affects interpretation and does not invalidate the MR framework.

To our knowledge, this study is the first to conduct a GWAS using lone AF as the phenotype. Compared to common AF, the genetic predisposition to lone AF is thought to involve a more significant contribution from pathogenic rare variants than common polymorphisms. However, genotyping studies have also indicated that common variants play a critical role in the development of lone AF. Choi et al. analyzed both monogenic and polygenic contributions to AF risk using UKB data [28]. Their findings revealed a strong association between the polygenic risk score and AF risk, whereas monogenic loss-of-function variants showed no significant relationship with AF risk for any gene, except TTN. Similarly, in this study, the heritability estimate from the GWAS was higher for lone AF than for common AF, suggesting that polygenic common variants contribute substantially to the development of lone AF.

A previous GWAS on AF has identified approximately 900 SNPs, and the latest large-scale cross-ancestry GWAS, which included 14,554 AF cases and 2,193,634 controls, reported 146 SNPs associated with AF [5]. In our study, most of the SNPs identified to be associated with lone AF have already been reported to be linked to AF in previous research. However, we identified two novel SNPs that had not previously been reported as associated with AF. One of these, rs74583115, is an intron variant in the FAT3 gene. The other, rs1038444414, is an intron variant in the PKP2 gene. While this gene is not known to be directly involved in cardiac pathogenesis, three other SNPs in this gene have been reported to be significantly associated with AF development.

The long-term clinical significance of lone AF has not been well-defined. Weijs et al. compared patients with lone AF to a healthy control group with normal sinus rhythm in a prospective study, and found that, over a follow-up period of approximately five years, the AF group had more than twice the incidence of cardiovascular disease [29]. Notably, although the overall incidence was low, events of stroke, HF, and myocardial infarction occurred only in the AF group. Also, in a Japanese cohort study involving 90,629 subjects, AF without traditional stroke risk factors was associated with more than a fourfold increased risk of stroke mortality compared to participants without AF [30]. The current MR analyses demonstrated that genetic predisposition to lone AF is strongly associated with increased risks of stroke and HF, suggesting that isolated AF is not likely a benign condition.

Common AF is considered to be associated with various adverse clinical outcomes, including stroke, HF, myocardial infarction, and cerebrovascular disease [2]. The causal relationship between AF and cardiovascular disease has been explored using MR methods. Hu et al., in a study utilizing GWAS data from six contributing studies including >1,000,000 individuals for instrumental variables, found a significant causal association between AF and HF, ischemic stroke, and cardiac death, but not with CAD [6]. A two-sample MR analysis on the relationship between AF and various cardiovascular disease subtypes suggested that the risk of cardioembolic stroke was more than double [31]. The results of the MR analysis on common AF in our study were consistent with those of previous studies. However, the odds of lone AF increasing the risk of stroke or HF were higher, which differs from previous clinical studies on lone AF [13,14]. Including all AF cases as a single phenotype may lead to the inclusion of SNPs associated with other phenotypes in GWAS results; using SNPs specific to lone AF as instrumental variables can help reduce such confounding, and the findings of this study appear to reflect this aspect.

In the current analysis, neither lone nor common AF was significantly associated with CAD. Although the sample size of the CARDIoGRAM dataset is smaller (overall 184,305) than other summary datasets used in our study, the effect did not lean towards increasing CAD, reinforcing the neutral impact of AF. A significant association with cardiac death risk was observed only for common AF. Although the effect size for cardiac death did not differ substantially between lone AF and common AF, the association reached statistical significance only for common AF, likely due to differences in the number of SNPs included. However, the overall effect size was small, and the significant association between AF and cardiac death was not replicated in sensitivity analyses using a two-sample MR method. Therefore, we think that the findings of this study do not support a strong association between AF and cardiac death.

This study highlights the polygenic effect of common variants in the development of lone AF and shows that AF is associated with more than twice the risk of HF and stroke compared to the general population even without other risk factors. However, our findings do not necessarily indicate that young patients with lone AF should undergo preventive interventions such as anticoagulation therapy. Importantly, the MR estimates reflect lifelong genetic susceptibility rather than short-term clinical risk, and should therefore be interpreted in the context of long-term disease risk rather than immediate clinical decision making. Nevertheless, proactive diagnosis and therapeutic interventions aimed at maintaining sinus rhythm, together with appropriate risk stratification and long-term monitoring strategies, may help reduce the absolute lifelong risk of AF-related complications in these patients.

### Limitations

Lone AF is typically defined as AF occurring at a young age without associated risk factors, though the definition varies across studies [32]. While the age limit for diagnosis has not been definitively established, many previous studies have commonly applied an age cutoff of <60 years, whereas others have used more lenient criteria that include older individuals. In the present study, we adopted an age cutoff of <60 years to align with the most widely used definition in the literature. Other exclusion criteria, such as severe obesity and heavy alcohol use, were operationally defined using directly measured variables (BMI and alcohol intake frequency), while comorbidities were defined based on ICD codes. As a result, the ascertainment of comorbidities based on ICD codes may not perfectly capture the underlying clinical conditions. The SNPs identified in this study’s GWAS for lone AF were mostly those already known to be associated with AF. Therefore, this study’s results are likely a refinement of AF-related SNPs, narrowing down to those more specific to isolated AF rather than presenting novel GWAS findings.

## 5. Conclusions

This GWAS using UKB data showed that common genetic variants significantly contribute to the development of lone AF. The MR analysis demonstrated that genetic predisposition to lone AF is associated with approximately a two-fold increase in the risk of stroke and HF. Both impacts were greater than those observed in common AF. However, lone AF was not associated with a higher risk of CAD. Also, its association with cardiac death was weak and not statistically significant. Our study findings suggest that AF, in the absence of other accompanying risk factors, may carry a significantly higher risk of stroke and HF than previously recognized. Further prospective studies are needed to determine their implications for clinical risk stratification and management.

## Figures and Tables

**Table 1:** Summary datasets used for MR analyses.

	Diagnosis	Case	Control	Prevalence	Ancestry	UKB Data Inclusion	Consortium
**Exposure**	AF	40,203	417,589	0.088	EUR		UKB
	Lone AF	4767	417,589	0.011	EUR		UKB
**Outcomes**	Coronary artery disease	60,801	123,504	0.330	EUR	No	CARDIoGRAMplusC4D
	Stroke	40,585	406,111	0.091	EUR	No	MEGASTROKE
	Heart Failure	68,408	1,286,331	0.050	EUR	No	GBMI
		47,309	930,014	0.048	EUR	Yes	HERMES
	Cardiac death	7563	211,229	0.035	EUR	No	FinnGen

Abbreviations: MR = Mendelian randomization; AF = atrial fibrillation; UKB = UK Biobank; and EUR = European.

**Table 2:** Novel genetic loci associated with lone AF identified in GWAS.

rsID	Chromosome	Position (GRCh37)	Gene (Nearest or Within)	Reference Allele	Alternative Allele	RAF	Beta	*p*-Value
rs74583115	11	92,140,057	FAT3 (intron)	C	G	0.126	0.002	2.6 × 10^−8^
rs1038444414	12	32,982,194	PKP2 (intron)	A	T	0.146	0.002	4.1 × 10^−8^

Abbreviations: AF = atrial fibrillation; RAF = risk allele frequency.

**Table 3:** GSMR estimates for the causal effects of lone AF and common AF on cardiovascular outcomes.

Outcome	Summary Dataset	Exposure	No. of SNPs	OR (95% CI)	*p*-Value	FDR Value
Stroke	MEGASTROKE	Lone AF	30	2.62 (2.14–3.22)	2.8 × 10^−20^	2.3 × 10^−19^
		Common AF	147	1.86 (1.69–2.04)	1.5 × 10^−36^	2.5 × 10^−36^
HF	GBMI	Lone AF	32	2.23 (1.90–2.60)	1.0 × 10^−23^	1.3 × 10^−22^
		Common AF	160	1.71 (1.59–1.84)	4.0 × 10^−48^	1.0 × 10^−47^
	HERMES	Lone AF	30	2.55 (2.14–3.04)	1.4 × 10^−25^	1.8 × 10^−24^
		Common AF	151	1.94 (1.79–2.11)	3.6 × 10^−57^	1.8 × 10^−56^
CAD	CARDIoGRAM	Lone AF	33	0.90 (0.73–1.10)	0.307	0.307
		Common AF	150	1.01 (0.92–1.12)	0.802	0.802
Cardiac death	FinnGen	Lone AF	30	1.32 (0.99–1.77)	0.059	0.185
		Common AF	148	1.28 (1.12–1.46)	3.0 × 10^−4^	3.8 × 10^−4^

Abbreviations: GSMR = generalized summary-data-based Mendelian randomization; AF = atrial fibrillation; SNP = single-nucleotide polymorphism; OR = odds ratio; HF = heart failure; CAD = coronary artery disease; and CI = confidence interval.

## Data Availability

The datasets used in this study are not publicly available, but they can be provided by the corresponding authors upon reasonable requests.

## Supplementary Materials

The following supporting information can be downloaded at: https://www.mdpi.com/article/10.3390/biomedicines14020413/s1, Figure S1: Scatter plots of generalized summary-data-based Mendelian randomization results showing the causal effect of common AF on stroke (A), HF in GBMI summary data (B), and HF in HERMES summary data (C), as well as the causal effect of common AF on CAD using CARDIoGRAM summary data (D), and cardiac death (E); Figure S2: Sensitivity analyses of the causal associations between genetically predicted common AF and cardiovascular outcomes using two-sample Mendelian randomization methods; Table S1: The definitions for inclusion and exclusion criteria; Table S2: SNP-level instrument strength for genetically predicted lone AF; Table S3: Two-sample MR analyses for the causal effect of lone AF; Table S4: Two-sample MR analyses for the causal effect of common AF; Table S5: Assessment of horizontal pleiotropy and heterogeneity in MR analyses.
