# Supplementary material for: Genetic Predisposition to Lone Atrial Fibrillation and the Causal Effect on Cardiovascular Diseases: A Mendelian Randomization Study
Source: Biomedicines. 2026 Feb 11;14(2):413. doi: 10.3390/biomedicines14020413 (PMC12938380; doi:10.3390/biomedicines14020413)
Supplement: Supplementary file 1 [file biomedicines-14-00413-s001.zip › biomedicines-4093054-supplementary.pdf]

Supplemental Materials to “Genetic Predisposition to Lone Atrial Fibrillation and the Causal Effect on Cardiovascular Diseases: A Mendelian Randomization Study”

Seunghwan Park [1,†], Hwajung Kim [2,3,†], Jieun Seo [1], Do Young Kim [3,4], Youmi Hwang [3,5], Sung-Hwan Kim [3,4], Kichang Lee [6,7], Wonil Chung [1,8,*] and Young Choi [3,4,*]

Supplemental Figures

Supplemental Figure S1. Scatter plots of generalized summary-data-based Mendelian randomization results showing the causal effect of common AF on stroke (A), HF in GBMI summary data (B), and HF in HERMES summary data (C), as well as the causal effect of common AF on CAD using CARDIoGRAM summary data (D), and cardiac death (E).

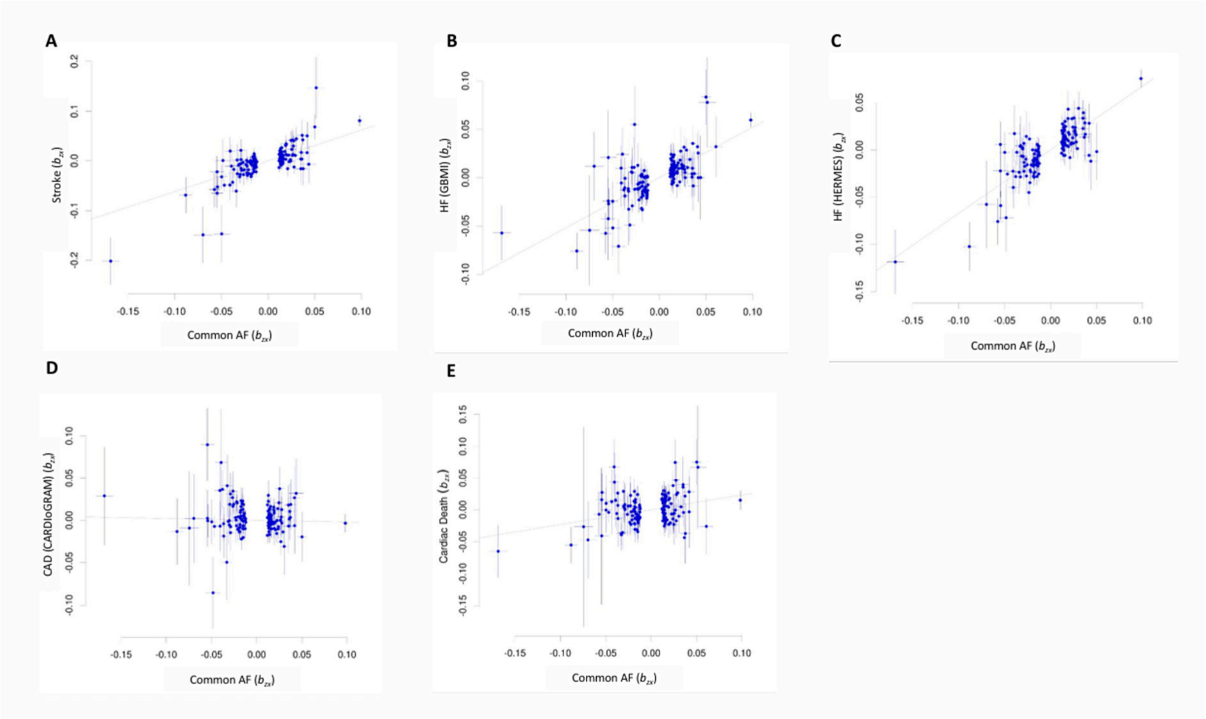

AF = atrial fibrillation; HF = heart failure; GBMI = Global Biobank Meta-analysis Initiative; HERMES = Heterogeneity and Remission of Metabolic Syndrome; CAD = coronary artery disease; CARDIoGRAM = Coronary Artery Disease Genome-Wide Replication and Meta-Analysis

Supplemental Figure S2. Sensitivity analyses of the causal associations between genetically predicted common AF and cardiovascular outcomes using two-sample Mendelian randomization methods.

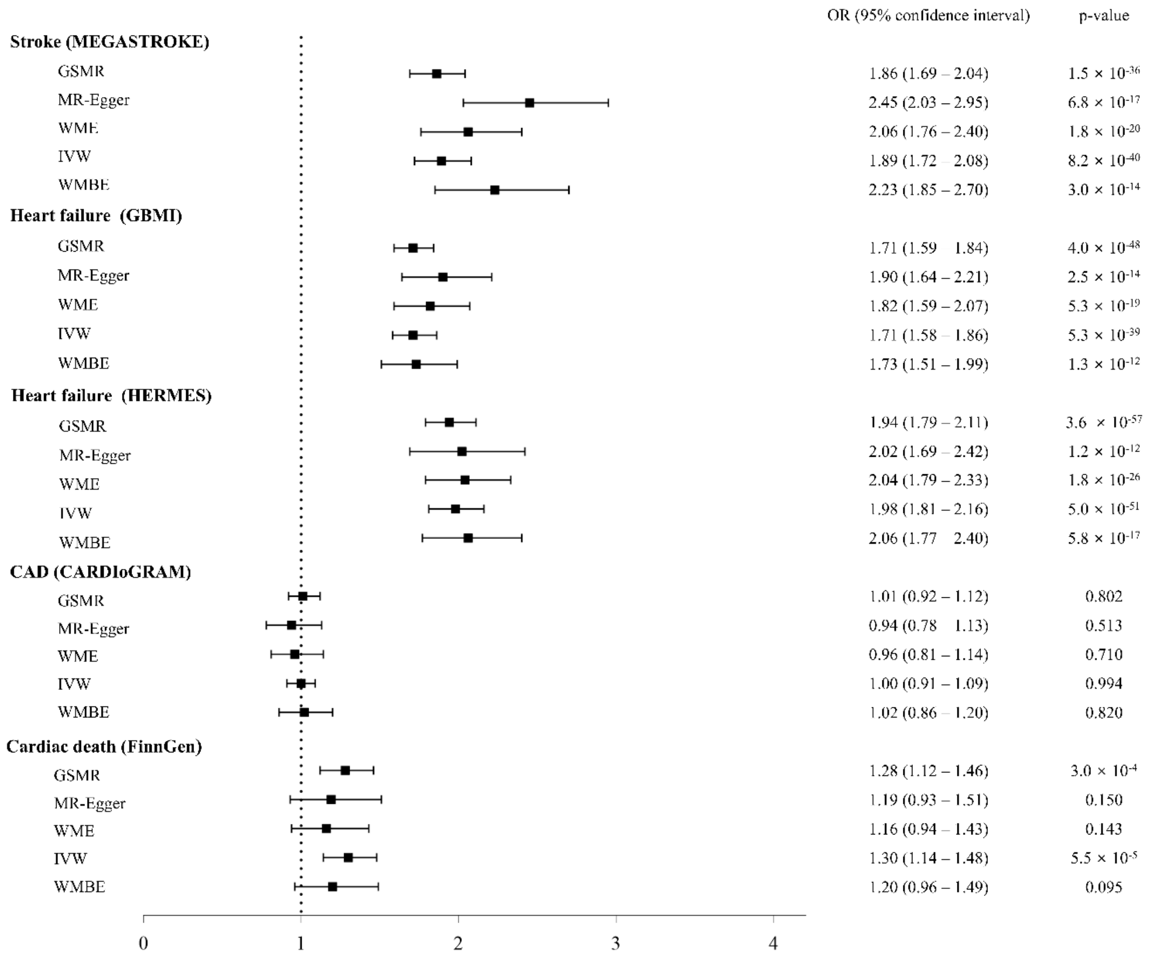

MR = Mendelian randomization; GSMR = generalized summary-based MR; WME = weighted median; WMBE = weighted mode-based estimator; CAD = coronary artery disease; OR = odds ratio

Supplemental Tables

Supplemental Table S1. The definitions for inclusion and exclusion criteria

Diagnostic criteria	**Definition (**ICD-10-CM code)
**Inclusion**	
Atrial fibrillation	I48
**Exclusion**	
Age ≥ 60 years	Age at the time of AF diagnosis
Hypertension	I10-I13, I15 and a prescription of antihypertensive medications
Diabetes mellitus	E10-E14
Coronary artery disease	I21-I23, I251, I252, I255
Valvular heart disease	I340-I342, I350-I352, I050-I052, I060-I062
Pulmonary disease	J42-44
Irreversible CMP	I420-I422, I424, I425
Hyperthyroidism	E059
Obstructive sleep apnea	E473
Severe obesity	BMI > 35 kg/m^2
Heavy alcoholics	Men: ≥5 drink per day or ≥15 drink per week
	Women: ≥4 drink per day or ≥8 drink per week

Supplemental Table S2. SNP-level instrument strength for genetically predicted lone AF.

SNP	Chromosome	Position (GRCh37)	Effect Allele	Other Allele	Effect allele frequency (EAF)	OR	SE	F statistics	R-square	Novelity
rs1443926	1	112,461,902	A	G	0.68	1.0017	0.0002	48.33	0.0026	X
rs6426987	1	154,815,257	C	A	0.65	0.9977	0.0002	90.65	0.0048	X
rs12144978	1	154,850,445	G	A	0.63	1.0016	0.0002	45.08	0.0024	X
rs72700103	1	170,180,343	A	C	0.88	0.9978	0.0004	39.24	0.0021	X
rs72700114	1	170,193,825	G	C	0.93	0.9964	0.0004	67.68	0.0036	X
rs56250774	1	170,632,384	A	G	0.82	0.9979	0.0003	49.36	0.0026	X
rs4590732	1	203,028,711	C	G	0.45	1.0015	0.0002	42.68	0.0023	X
rs2358891	2	175,558,804	G	A	0.76	1.0015	0.0003	29.72	0.0016	X
rs557785	4	111,462,203	A	C	0.08	1.0024	0.0004	32.90	0.0017	X
rs5861016	4	111,592,031	A	AT	0.30	1.0026	0.0002	110.03	0.0058	X
rs79041359	4	111,610,855	C	A	0.74	1.0016	0.0003	32.30	0.0017	X
rs62337205	4	111,650,325	A	G	0.95	1.0031	0.0005	35.68	0.0019	X
rs112599895	4	111,656,592	A	G	0.99	0.9893	0.0011	102.34	0.0054	X
rs114691030	4	111,675,514	G	C	0.97	0.9963	0.0007	30.95	0.0016	X
rs77668866	4	111,687,977	C	T	0.91	1.0022	0.0004	31.02	0.0016	X
rs6843082	4	111,718,067	G	A	0.19	1.0067	0.0003	537.11	0.0277	X
rs13105878	4	111,718,147	C	A	0.90	1.0022	0.0004	33.00	0.0017	X
rs3853445	4	111,761,487	T	C	0.74	1.0032	0.0003	154.45	0.0081	X
rs4834450	4	111,782,769	A	G	0.05	0.9971	0.0005	30.89	0.0016	X
rs3853438	4	111,810,048	G	A	0.31	0.9981	0.0002	55.69	0.0029	X
rs17513625	4	111,848,270	G	A	0.98	0.9937	0.0008	70.46	0.0037	X
rs374582	4	111,849,422	G	A	0.39	1.0016	0.0002	49.11	0.0026	X
rs11773845	7	116,191,301	C	A	0.41	0.9978	0.0002	91.38	0.0048	X
rs4744411	9	97,689,045	G	A	0.58	0.9987	0.0002	33.81	0.0018	X
rs3740422	10	103,565,960	G	C	0.65	1.0015	0.0002	39.27	0.0021	X
rs3781295	10	104,140,602	G	A	0.63	0.9986	0.0002	32.84	0.0017	X
rs11598047	10	105,342,672	A	G	0.85	0.9977	0.0003	50.90	0.0027	X
rs74583115	11	92,140,057	C	G	0.87	0.9981	0.0003	30.53	0.0016	O
rs3765618	11	128,769,876	C	G	0.91	0.9968	0.0004	62.75	0.0033	X
rs35628018	12	32,982,194	A	AT	0.85	0.9982	0.0003	30.06	0.0016	O
rs7312625	12	114,799,974	G	A	0.26	0.9981	0.0003	53.61	0.0028	X
rs445754	14	23,863,802	G	T	0.77	0.9981	0.0003	47.72	0.0025	X
rs8005490	14	35,184,541	T	C	0.41	1.0013	0.0002	33.32	0.0018	X
rs140185678	16	2,003,016	G	A	0.97	0.9966	0.0006	29.40	0.0016	X
rs67329386	16	73,048,367	C	T	0.83	0.9965	0.0003	126.97	0.0067	X
rs62055084	16	73,097,845	C	T	0.73	1.0017	0.0003	42.42	0.0022	X

OR = Odds Ratio; SE = Standard error

Supplemental Table S3. Two-sample MR analyses for the causal effect of lone AF

Outcome (Dataset)	Methods	OR (95%CI)	p-value	FDR value
Stroke (MEGASTROKE)	MR EGGER	6.51 (4.42 - 9.57)	2.9E-10	1.5E-09
	WME	3.28 (2.52 - 4.27)	8.6E-19	4.3E-18
	IVW	2.78 (2.22 - 3.48)	3.5E-19	8.8E-19
	WMBE	3.37 (2.48 - 4.56)	1.3E-08	3.3E-08
Heart failure (GBMI)	MR EGGER	3.12 (2.18 - 4.46)	3.1E-08	7.8E-08
	WME	2.40 (1.92 - 3.00)	1.5E-14	2.5E-14
	IVW	2.27 (1.86 - 2.77)	6.0E-16	1.0E-15
	WMBE	2.53 (2.00 - 3.21)	8.9E-08	1.5E-07
Heart failure (HERMES)	MR EGGER	3.71 (2.62 - 5.26)	4.9E-08	8.2E-08
	WME	2.72 (2.13 - 3.47)	1.4E-15	3.5E-15
	IVW	2.69 (2.26 - 3.20)	6.6E-29	3.3E-28
	WMBE	2.90 (2.25 - 3.73)	4.0E-09	2.0E-08
CAD (CARDIOGRAM)	MR EGGER	1.22 (0.79 – 1.86)	0.376	0.376
	WME	0.96 (0.73 – 1.26)	0.749	0.749
	IVW	0.86 (0.69 – 1.06)	0.158	0.158
	WMBE	1.01 (0.77 – 1.31)	0.961	0.961
Cardiac death (FinnGen)	MR EGGER	1.36 (0.90 - 2.04)	0.150	0.188
	WME	1.22 (0.86 - 1.71)	0.263	0.329
	IVW	1.25 (1.02 - 1.54)	0.034	0.043
	WMBE	1.20 (0.87 - 1.65)	0.275	0.343

OR = Odds Ratio; CI = Confidence interval; FDR = false discovery rate; WME = weighted median; IVW = inverse variance weighted; WMBE = weighted mode-based estimator

Supplemental Table S4. Two-sample MR analyses for the causal effect of common AF

Dataset (Outcome)	Methods	OR (95%CI)	p-value	FDR value
Stroke (MEGASTROKE)	MR EGGER	2.45 (2.03 – 2.95)	6.8E-17	3.4E-16
	WME	2.06 (1.76 – 2.40)	1.8E-20	4.5E-20
	IVW	1.89 (1.72 – 2.08)	8.2E-40	2.1E-39
	WMBE	2.23 (1.85 – 2.7)	3.0E-14	7.5E-14
Heart failure (GBMI)	MR EGGER	1.90 (1.64 – 2.21)	2.5E-14	6.3E-14
	WME	1.82 (1.59 – 2.07)	5.3E-19	8.8E-19
	IVW	1.71 (1.58 – 1.86)	5.3E-39	8.8E-39
	WMBE	1.73 (1.51 – 1.99)	1.3E-12	2.2E-12
Heart failure (HERMES)	MR EGGER	2.02 (1.69 – 2.42)	1.2E-12	2.0E-12
	WME	2.04 (1.79 – 2.33)	1.8E-26	9.0E-26
	IVW	1.98 (1.81 – 2.16)	5.0E-51	2.5E-50
	WMBE	2.06 (1.77 – 2.40)	5.8E-17	2.9E-16
CAD (CARDIOGRAM)	MR EGGER	0.94 (0.78 – 1.13)	0.513	0.513
	WME	0.96 (0.81 – 1.14)	0.710	0.710
	IVW	1.00 (0.91 – 1.09)	0.994	0.994
	WMBE	1.02 (0.86 – 1.20)	0.820	0.820
Cardiac death (FinnGen)	MR EGGER	1.19 (0.93 – 1.51)	0.150	0.185
	WME	1.16 (0.94 – 1.43)	0.143	0.179
	IVW	1.30 (1.14 – 1.48)	5.5E-05	6.9E-05
	WMBE	1.20 (0.96 – 1.49)	0.095	0.119

OR = Odds Ratio; CI = Confidence interval; FDR = false discovery rate; WME = weighted median; IVW = inverse variance weighted; WMBE = weighted mode-based estimator

Supplemental Table S5. Assessment of horizontal pleiotropy and heterogeneity in MR analyses.

Exposure	Outcome	Dataset	HEIDI outliers (n)	MR-PRESSO outliers (n)	Cochran's Q (p)
Lone AF	Stroke	MEGASTROKE	1	2	7.7E-05
	Heart failure	GBMI	0	1	4.7E-04
		HERMES	1	2	4.1E-04
	CAD	CARDIoGRAM	1	0	0.091
	Cardiac death	FinnGen	0	0	0.341
Common AF	Stroke	MEGASTROKE	3	2	8.0E-04
	Heart failure	GBMI	5	1	3.5E-07
		HERMES	2	1	1.2E-07
	CAD	CARDIoGRAM	5	2	0.011
	Cardiac death	FinnGen	3	1	0.009

HEIDI-outlier filtering was applied within the GSMR framework, and MR-PRESSO was used as a sensitivity analysis where applicable. Heterogeneity across genetic instruments was assessed using Cochran’s Q statistic derived from inverse-variance weighted (IVW) MR analyses. Abbreviations : CAD = coronary artery disease; AF = atrial fibrillation
